# Assessing the Risk of Stress in Organizations: Getting the Measure of Organizational-Level Stressors

**DOI:** 10.3389/fpsyg.2019.02776

**Published:** 2019-12-20

**Authors:** Stephen Wood, Valerio Ghezzi, Claudio Barbaranelli, Cristina Di Tecco, Roberta Fida, Maria Luisa Farnese, Matteo Ronchetti, Sergio Iavicoli

**Affiliations:** ^1^School of Business, University of Leicester, Leicester, United Kingdom; ^2^Department of Psychology, Sapienza University of Rome, Rome, Italy; ^3^Department of Occupational and Environmental Medicine, Epidemiology and Hygiene, Italian Workers’ Compensation Authority (INAIL), Rome, Italy; ^4^Norwich Business School, Faculty of Social Sciences, University of East Anglia, Norwich, United Kingdom

**Keywords:** risk for work-related stress, organizational-level stressors, demand–control–support theory, role conflict and clarity, employee stress and well-being, psychometric isomorphism, Management Standards Indicator Tool

## Abstract

Great Britain’s Health and Safety Executive (HSE) developed the Management Standards Indicator Tool to help organizations to assess and monitor organizational risks of work-related stress through surveying employees about the psychosocial risks for stress in their jobs. The use of employee-level data for deriving an organizational-level measure of psychosocial risks assumes that the constructs have equivalent meanings at different levels. However, this isomorphic condition has never been tested and this study fills this gap. Using data collected by the Italian Workers’ Compensation Authority (INAIL) from 66,188 employees nested in 775 organizations, we demonstrate that the organizational-level measure representing the seven dimensions of the Management Standards Indicator Tool is equivalent, though not identical, to the individual-level measure. This implies that the organizational level is not a mirror of the aggregation of the individual level, and that the risk of work-related stress in an organization may derive not simply from bottom-up processes, but may be generated by top-down influences (e.g., organizational policies). Interventions may then be meaningfully targeted at the organizational level in the expectation that they will reduce the risk of work-related stress among the entire workforce, the valid measurement of which can be performed through the HSE’s Management Standards Indicator Tool.

## Introduction

Surveying employees about their jobs is a core method for obtaining information on job-related stressors. However, Great Britain’s Health and Safety Executive (HSE) has developed such a survey with the aim of using it to also measure organizational-level stressors, the intention being to derive these measures through an aggregation method, rather than asking employees directly about organizational-level stressors (such as organizational policies and norms about workloads or events such as downsizing or tight staffing levels). In multilevel terminology, it relies on the direct consensus approach to composition, as opposed to the referent-shift approach (Chan, 1998). The problem this paper addresses is whether such a procedure can yield a valid measure of organizational-level stressors as its architects intended, or whether use of the data must be confined to measuring job-level stressors. We thus report research using multilevel factor analysis of data collected by the Italian Workers’ Compensation Authority (INAIL)^1^ to test whether the organizational- and job-level measures have equivalent meanings; that is, we assess the isomorphism between the two. The individual-level measures are based on Great Britain’s HSE’s questionnaire for assessing job-level stressors, known as Management Standards Indicator Tool.

We first introduce the Management Standards Indicator Tool, including the theoretical and policy context in which it was developed, and its potential utility and nature. We then discuss the past evaluations of it and our approach to testing isomorphism, the various ways in which isomorphism may occur and hence be expected, and the potential diverse types of isomorphism that may be consistent with the data, concluding with the hypotheses we test in the light of this diversity. We then report the nature of the study, the methods of analysis, and the results of our study. We conclude this paper by drawing out the theoretical and practical implications of the study and its strengths and weaknesses.

The paper meets calls to ensure that in multilevel research constructs generalize across levels and researchers ensure precision in their levels of analysis and theorization (Rousseau, 1985; Morgeson and Hofmann, 1999). In so doing it is a significant response to calls from Bliese and Jex (2002) and Probst (2010) encouraging researchers to consider multiple levels of analysis in the stress area. These are being made on the basis that stressors could reside in levels other than the job level, which has dominated work-related stress research, including the organizational, team, and personal levels. The precise importance of these can only be ascertained when we have valid measures of all these levels but there are currently sufficient indications that the organizational-level factors play a role in employees’ stress, as well as creating collective emotional states and moods (e.g., Totterdell et al., 1998; Bartel and Saavedra, 2000; Bakker et al., 2006), to justify focusing on the organizational level as we do here. More specifically, our paper contributes to the organizational stress field in several ways. First it offers, by using a multilevel construct validation approach, an organizational measurement tool that can be used by researchers to assess the effect of organizational-level stressors on individual stress and other employee outcomes, and by practitioners to gauge the level of organizational-level stressors in organizations and tailor interventions to affect these. Second, it examines the extent to which the seven factors in the HSE’s Management Standards Indicator Tool are both discrete at either the organizational or individual level and whether they represent the most parsimonious measures at each level. Third, it provides an exemplar application of the framework for testing psychometric isomorphism developed by Tay et al. (2014) to evaluate the similarity among individual- and organizational-level factors. Fourth, it offers evidence of the external validity of the organizational-level psychosocial risk factors for work-related stress by evaluating their differences across industrial and economic sectors and their associations with organizational-level indicators of work-related stress.

### Assessing the Risk of Stress in Organizations

The director of a hospital engaged one of the authors to help confront a problem of burnout among nurses. Having stressed that the context was an especially stressful one, and the burnout was not just the result of day-to-day pressures of the job, the director nonetheless requested that s/he intervene through a program of one-to-one counseling. This example is not atypical of the commonplace mismatch in organizational contexts between interventions and the problem being addressed. Underlying such mismatches is a lack of appreciation of the important distinctions in stress theory. First, the fundamental one is between stress and stressors, between the state of stress and what induces it. Second, that between types of stressors: most significantly between individual-level, job-level, and organizational-level stressors. Third, that between interventions targeted at individuals, jobs, or the whole organization. As the hospital manager’s case illustrates the tendency is to focus on the effects of stress and interventions aimed at reducing individuals’ stress levels, as if the dominant source of stress is internal to the individual or at best, if external, the task is to increase coping or resilience.

Occupational psychologists and public health and safety bodies have drawn attention to such tendencies and sought to develop richer perspectives. For example, Kompier et al. (2000) gauged that psychosocial work characteristics are too often neglected as organizations focus more on the effects of the stress than on its causes. Associated with this, others have pointed to the fact that, where interventions have been made, they have predominantly been stress-management programs aimed at individuals – for example, the provision of employee assistance programs, exercise classes, and training in coping skills or time management (Cox et al., 2007).

A core element of the research on work-related stress has, nonetheless, focused on externally generated stressors, and particularly the job-level stressors to the neglect of personal- and organizational-level stressors. A review of the 197 studies (based on a systematic search by ourselves)^2^ that included the term “organizational stressors” revealed that the term is being used as a synonym for one or other of occupational, psychosocial, job, or work stressors. Allied to this has been a concentration on job design as a key policy instrument, complemented by supportive leadership. Reflecting this, developments in measuring stressors have concentrated on job characteristics. Tabanelli et al. (2008) identified 33 such instruments, the majority of which use questionnaires as opposed to observational methods. These include the Job Content Questionnaire (JCQ; Karasek et al., 1998) founded on the demands and control theory of stress, and Hackman and Oldham’s (1980) Job Diagnostic Survey, all developed in the last century.

Subsequently, it was public bodies such as the Great Britain’s HSE and the United States’ National Institute of Occupational Safety and Health (NIOSH) who did most to emphasize the distinction between job-level and organizational-level stressors. In the early 2000s, such public bodies encouraged organizations to focus more on the causes of work-related stress than the individuals’ stress level. For example, the United States’ National Institute of Occupational Safety and Health [NIOSH] (2002) highlighted the potential role of organizational factors – including downsizing, new technologies, skill-mixes, and flexible employment contracts – on health and safety and on the need for intervention research “targeting organizational practices and policies that may protect worker safety and health” (National Institute of Occupational Safety and Health [NIOSH], 2002, p. vii). In these terms, organizational-level stressors are potentially common to all employees, that arise from a mixture of policy, top management leadership style, culture, legislation, bottom-up emergent processes, and events such as downsizing and recessions. Great Britain’s HSE went further than simply drawing attention to such factors and urged practitioners “to focus on organizational-level issues,” arguing this principally on the basis that they “have the potential to impact on groups and large numbers of employees, rather than individual employees [*per se*]” (Health and Safety Executive [HSE], 2009, p. 6). Subsequently, LaMontagne et al. (2007) have demonstrated that organizational-level interventions concerned with preventing stress are more effective than interventions targeted directly at individuals.

Great Britain’s HSE developed the Management Standards Indicator Tool as part of their promotion of the importance of organizational-level stressors. It involves a questionnaire for assessing job-level stressors through individual employees’ assessing their experience of key job characteristics, the demands, resources and support they have, the design of which reflects the focus of Karasek’s, and others’ measures. But the intention behind the instrument was that the individual-level data could be used to create measures of organizational-level stressors and not simply job-level ones. Seven stressors were included in the tool and were rooted in the extended Karasek’s model that focused on high demands, low control, and limited support as stressors (Johnson and Hall, 1988; Karasek and Theorell, 1990). Previous validation exercises of the HSE’s Management Standards Indicator Tool have tested if the observed data conform to the seven distinct dimensions at the individual level (e.g., Edwards et al., 2008; Toderi et al., 2013) and have found it did, but this only confirms that the instrument may yield a valid measure of job-related stressors. They have not tested if the measure has the same psychometric properties across both the individual and organizational levels and hence can fulfill HSE’s purpose of measuring organizational-level risk factors for work-related stress. No study has demonstrated the capacity of this or any of the other instruments in the area to capture risk factors for work-related stress at the organizational level using individual-level data.

The study we report in this paper is aimed at filling this gap, by assessing whether a meaningful measure of organizational-level stressors can be derived from individual reports of the psychosocial risk factors associated with their jobs and working environment, through testing the similarity or isomorphism between organizational- and individual-level constructs. While this could be done with data collected using any of a number of instruments, we use data collected using the HSE’s Management Standards Indicator Tool. Our reasons for this are first because the distinctiveness of the HSE’s program included the aim of achieving a measure of organizational-level stressors and, until the validity of this approach for measuring organizational-level stressors has been established, an important element of the evidence base for its widespread adoption is missing. Second, its core elements include stressors concentrating on the intrinsic aspects of jobs that are covered in other significant instruments. Third, it has been adopted outside of the United Kingdom, and notably in Italy as part of the toolkit developed by INAIL for helping organizations fulfill their statutory obligations to monitor the stress risks of employees. Fourth, we were given access to the dataset that has been developed by INAIL which is a distinctive, if not unique, and includes data from over 750 organizations involving more than 66,000 employees who completed the Italian version of the Management Standards Indicator Tool.

### Theoretical Assumptions, Nature, and Potential Utility of the Management Standards Indicator Tool

The novelty of the HSE’s approach lies in its aim of providing an instrument to assess psychosocial risks across the organization and not just individual-level risk factors (Cox et al., 2000; MacKay et al., 2004; Cox et al., 2007). The Management Standards Indicator Tool, which fulfills this aim, was first intended to be a measurement tool to help employers, in conjunction with their workforces, to monitor the psychosocial risk factors in their organizations and to identify the key stressors in their organizations, and to design and evaluate targeted interventions at the organizational level. It can similarly be used by public bodies to collect systematic datasets for developing thresholds about the risks for work-related stress across the economy or sectors, which will aid benchmarking and the setting of realistic targets for organizations, as INAIL has been doing since 2011. Equally important, it is also relevant for researchers, enabling them to incorporate organizational-level factors into the analysis of stress. Without instruments of this kind, we cannot test the very assumption that underlies the promotion of the tool: that organizational-level stressors play a role in employee stress.

This assumption is the core of the theory underlying the instrument, which we can identify as having three main elements. First, the theory distinguishes four main levels of stressors: personal, job, group, and organizational. Second, it assumes that employees stress levels can be affected by all four types of stressors, and though the focus among occupational psychologists has been on the job level, the organizational level may yet be crucial. Third, the assumption is that the dimensions typically associated with the job level may be applicable to the organizational level. Specifically, that organizations can be characterized in terms of the demands, resources, and supports conceptual framework; that is, in terms of how demanding, resource-limiting, and unsupportive they are. This reflects longstanding concepts in organization theory. For example, the notion of the bureaucratic organization implies a hierarchical and rule-based system which may limit autonomy and overcontrol people. Human relations theory drew attention to the limited autonomy in organizations designed according to Taylorist and McGregor’s (1960) Theory X principles, or the contrasting Theory Y design centered on fostering what Likert (1967) called supportive relationships. The concepts of high-involvement or high-commitment management (Lawler, 1986; Walton, 1995) and their derivative, the high-performance work system (Appelbaum et al., 2000), are founded on the notion that organizations can be characterized by the extent to which they foster employee autonomy, give employees a sense of direction, and create participative and supportive environments.

Furthermore, though not mentioned in accounts of the Management Standards Indicator Tool, it is significant that at the time of its development increasing recognition was being given to the possibility that collectives have moods and can be characterized as stressful (Totterdell et al., 1998; Bakker et al., 2006). The theory thus can be extended to such collective concepts, so that organizational-level stressors should figure in explanations of the “stressful organization.”

Applying such theoretical propositions, the core instrument of the Management Standards Indicator Tool, the employee questionnaire, centers on characteristics of the job and work context which constitute psychosocial risk factors for work-related stress. The choice of such dimensions was the result of extensive research by or on behalf of the HSE and the involvement of key stakeholders (MacKay et al., 2004). The instrument reflects the emphasis on job demands, job control, and support in the demand–control theory of Karasek (1979), which was later extended to include managerial supportive management (Johnson and Hall, 1988; Karasek and Theorell, 1990). Initially specified in HSE guidelines in 2001, the taxonomy includes: Demands, Control, Support, Relationships at work, Role clarity, Support for Change, and Culture. In developing the standards, culture was omitted as “it underpins the […] others” (MacKay et al., 2004, p. 95). In the Management Standards Instrument, the two significant types of support (that from line management and colleagues) are separated so management and peer support form separate factors, as they are conceived as distinct sources of social support by organizational actors (Cousins et al., 2004; Edwards et al., 2008). The seven stressors included in the instrument were considered by experts to be common to the main existing taxonomies (MacKay et al., 2004). The methodology is based on asking employees about the seven stressors as they affect them, using a 35-item questionnaire. The final version was derived on the basis of an exploratory factor analysis of 3,147 responses to a 100-item questionnaire, as items with low loadings (<0.50) were omitted and a seven-factor model fitted the data (Cousins et al., 2004).

Of the other instruments available, the Management Standards Instrument is most similar to the JCQ, which is based on the Karasek’s model and measures respondents’ work tasks using the high-demand/low-control/low-support model of job strain (Karasek et al., 1985), and to the Hackman and Oldham’s (1980) job characteristics model, with its emphasis on skill variety, task identity, task significance, autonomy, and feedback. Such instruments contrast with others in the review by Tabanelli et al. (2008), for example, those based on the effort–reward model (e.g., Siegrist et al., 2004), or those that include items beyond psychosocial stressors, such as coping strategies or stress reactions. The Copenhagen Psychosocial Questionnaire (COPSOQ-II, Kristensen et al., 2005), for example, includes individual’s health, well-being, personality, coping style, and self-esteem. The General Nordic Questionnaire (QPS NORDIC, Lindstrom et al., 2000) measures psychological/social factors as potential determinants of motivation, health, and wellbeing, and also includes individual’s competence, preference for challenge, work motives, and private-life interactions.

Advocacy of the Management Standards Indicator Tool for both research and management purposes also contrasts with the argument that the factors affecting stress will be specific to certain contexts and particularly will vary by occupation (Sparks and Cooper, 1999) which, taken to its extreme, implies that instruments must be tailored to specific occupational and organizational contexts. However, van Veldhoven et al. (2005) tested whether the correlations between stressors (demands, control, and support) and stress indicators (commitment, fatigue, and task satisfaction) varied by industry and found this was not the case.

Van Veldhoven et al. (2005) also tested alternative factorial structures of a measure of stressors comparing them with the three-factor model consistent with the Karasek’s (1979) tripartite architecture, and they found that models with more factors (e.g., by separating out types of job demands or including Hackman and Oldham’s skill utilization) fitted the data better. This suggests that any tests of the factor structure of the Management Standards Indicator Tool, including of the isomorphism between the individual-level and organization-level factors, should compare models with differing factor structures.

Our study is based on a set of questions that does not cover all the items included in the available instruments for collecting data, so we cannot directly compare the Management Standards Indicator Tool with these. We can and will test to see if more parsimonious models do not fit worse than a seven-factor model. Tests for isomorphism could of course be done with any dataset that includes stressors, and our results may be taken to have relevance to those that concentrate on intrinsic factors, but in omitting particular economic factors such as wages and job security, their relevance does not extend to all stressors, as, for example, outlined in Warr’s (2007, pp. 81–110) vitamin model. We could, for example, find no isomorphism between the measures derived from our tests, but this might not rule out isomorphism between measures based on more economic items.

### Previous Evaluations of the Management Standards Indicator Tool

Past evaluations of the construct validity of the Management Standards Indicator Tool have only assessed the seven-factor structure at the individual level. Using confirmatory factor analysis, three studies confirmed that the pattern of covariances between the 35 items conformed to the hypothesized seven-factor model (Edwards et al., 2008; Rondinone et al., 2012; Toderi et al., 2013). From such results, the researchers have concluded, in the words of Edwards et al. (2008, p. 105) that the HSE framework provides “a psychometrically robust instrument” that can be used “as a reliable and representative measure of work-related *stress* (*our emphasis*).” There are, however, two problems with this.

First, the Management Standards Indicator Tool measures psychosocial risk factors for work-related stress and not stress *per se*, so one can draw conclusions about the risk of stress in an organization using the tool but not the actual level of stress. Acknowledging the stressor–stress distinction avoids using the terms work-related stress, risk for stress, and stressors interchangeably when describing the Management Standards Indicator Tool.

Second, the assumption behind Edwards et al.’s conclusion is that the successful single-level factor analyses of individual-level data mean that one can use the tool for deriving organizational-level measures of psychosocial risks for work-related stress. However, in so doing, it is assumed that the tool has the same psychometric properties and the constructs have equivalent meaning at different levels of analysis, a situation known as psychometric isomorphism, defined as “similarity or one-to-one correspondence between two or more elements” (Bliese et al., 2007, p. 553) across two (or more) levels of analysis. In the Management Standards Indicator Tool case, it is assumed that the items reflect similar or identical underlining constructs across the individual and organizational levels. However, it may be that certain items represent organizational-level psychosocial risk factors stronger than they do at the individual level, or vice versa. For example, timing control items (Jackson et al., 1993) may reflect the control dimension more strongly at the organizational than at the individual level. The single-level approach, however, only yields a measure of the level of stressors associated with an individual’s job and work environment, so the past factor analytic studies of the Management Standards Indicator Tool are not sufficient to conclude that the measure meets its purpose of providing the basis for assessing risk factors for work-related stress at the organizational level. Assessing if individual responses can be used to create a meaningful organizational-level measure, and whether such a measure can have the same meaning as that at the individual level, requires reframing research questions within a multilevel perspective.

### Evaluating the Psychometric Isomorphism of the Management Standards Indicator Tool

Fulfilling the aim behind the architects of the Management Standards Indicator Tool of using individual-level data to create measures of organizational-level stressors requires, in multilevel terminology, an elemental composition process (Chan, 1998). This approach is referred as the direct consensus method in contrast to the reference-shift approach, which would yield an organizational-level measure on the basis of asking individuals about organizational-level stressors. Much of the discussion of the direct consensus approach has concentrated on team characteristics as emergent properties from within the group, that is, bottom-up processes (Tay et al., 2014). But organizational-level phenomenon may not be solely, or even predominantly, emergent from lower levels as they may be shaped by processes of leadership and policy making, that is, top-down processes (Tay et al., 2014).

Regardless of the generative source of the organizational-level phenomenon, multilevel factor analytic approach can be used to assess the validity of developing an organizational-level factor from the individual-level data based on the Management Standards Indicator Tool. It tests the factor structure of the Tool at both individual and organizational levels and it enables us to answer two related questions: (1) whether, when using the individual-level data to construct the higher level construct, the individual and organizational phenomena are similar, and (2) what the nature of any similarity between levels is. Testing for the similarity of the constructs between the two levels avoids the danger of the ecological fallacy, that is, inferring characteristics of organizational-level phenomena from results at the individual level (Rousseau, 1985; Morgeson and Hofmann, 1999).

Tay et al. (2014) provide a taxonomy of the types of psychometric isomorphism, which should be viewed as hierarchically ordered isomorphic conditions. First, they distinguish between configural and metric isomorphism which, in turn, can be further differentiated between weak and strong conditions. Weak configural isomorphism occurs when the same number of latent dimensions is found across levels, but the pattern of loadings differs. Strong configural isomorphism is assumed when the items of a scale load onto the same factor at the lower and the higher level and the pattern of fixed loadings are the same (e.g., job control items load only on the control factor at both levels). Weak metric isomorphism occurs when the rank ordering of factor loadings is congruent across levels, but the loadings themselves are not exactly the same (e.g., role clarity item 1 is the item with the highest factor loading at both levels but the magnitude of the loadings is not the same). Finally, strong metric isomorphism is reached when the magnitude of factor loadings is the same at both the lower and higher level (e.g., role clarity items load onto the referent latent variable in exactly the same way across levels).

A lack of configural isomorphism, strong or weak, would indicate that individual-level constructs are not generalizable to the organizational level. It may reflect that the factor structure differs between the two levels and a referent shift approach is required to capture the organizational stressors. Or it may be the result of a poorly fitting model which could mean that organizational stressors are not an identifiable phenomenon or, less dramatic, that these differ between groups within the organization, and ultimately that occupational differences are more significant than organizational factors for stress levels. A lack of strong metric isomorphism would imply that a given individual construct is not identical to its organizational counterpart. Such a weak isomorphism might be construed as strengthening the argument for attending to organizational stressors. In contrast, a strong one might mean that they are totally emergent from the individual phenomenon or alternatively that the latter totally reflect the organizational level, e.g., they are determined by policies and cultures created by top managers or founding fathers.

The precise meaning of isomorphic concepts and their ontological status is, however, a matter of theory, as Tay et al. (2014, p. 80) imply, as it is also “how they come to be.” The influences on the development of individual- and organizational-level constructs, or the processes through which they are determined may vary between the two levels, but this does not necessarily imply that they cannot be isomorphic.

### Isomorphism Through Combinations of Top-Down and Bottom-Up Processes

The processes that affect both the individual- and the organizational-level constructs may in fact include top-down processes, such as leadership and organizational policies specifically on job design, or bottom-up individual-level influences, such as perceptions of managers’ personal styles, task exigencies, and interdependencies. For example, in the case of control, there may be some similarity in the level of autonomy of jobs in an organization that reflects a common approach to the design of jobs, different from that in another organization. While, for instance, a research assistant in a university has less autonomy than a professor, and an assembly worker has less autonomy than a manager in a manufacturing plant, the underlying orientation in the university will be to give greater discretion to all employees than would be the case in the manufacturing plant. Similarly, some organizations will have a generalized norm of being especially demanding of their employees – for example, they might be expected to complete tasks before going home or to work outside normal hours – while another organization is less demanding. In the case of support, the overall approach of the organization may be to follow Likert’s (1967) principle of supportive relations, so that the majority of employees receive strong managerial support. As these examples suggest, organizational policies and practices are often designed to foster common ways of behaving, and to equalize workloads and discretion levels. We might therefore expect some similarities among employees’ experiences within a given organization, but we do not expect that individual responses are interchangeable within the organization (Bliese, 2000).

Events such as reductions in demand as in recessions or increasing costs in inflationary times may also change or create new organizational stressors. The literature on downsizing illustrates the effects that restructuring can have on well-being through increasing demands and reducing autonomy (Moore et al., 2004; Quinlan and Bohle, 2009). Research on the consequences on the 2008 recession also reveals such effects (Burke et al., 2015; Giorgi et al., 2015; Chaves et al., 2018). Often these changes are permanent and not reversed when conditions improve and may be intensified by further such actions. Significantly there is research showing that the stress of those that remain following a layoff program may be as great as those actually laid-off (Kivimäki et al., 2000; Campbell-Jamison et al., 2001).

Bottom-up processes may also contribute to an organizational-level risk factor for work-related stress. For example, contagion effects may result in commonalities between individuals’ evaluations of their job, such as when one person’s perceptions of excessive demands or disquiet with a non-supportive manager or peer are transmitted to others. Alternatively, there may be similarities in individuals’ crafting of their jobs.

These examples of top-down and bottom-up processes imply that we might expect some form of isomorphism between the individual- and organizational-level constructs that we derive from data collected from individuals about the psychosocial risk factors for work-related stress associated with their jobs. Nonetheless, the extent to which these constructs have identical meaning across levels needs testing, since there are scenarios where top-down and bottom-up processes differ substantially between the two levels (Tay et al., 2014). For example, top-down processes may be dominated by external forces, such as when senior managers are closely controlled by shareholders or the organization faces strong competitive or budgetary constraints, whereas the culture at local levels may be very diverse and reflect differences in tasks and histories. We thus test that:

Hypothesis 1: *Data on the 35 items conforms to a seven-factor model both at the individual level and at the* organization*al level, positing* (Model 1) *the same pattern of fixed and free factor loadings across levels of analysis.*

If this hypothesis of strong configural isomorphism is not rejected, then we hypothesize that factor loadings across individual and organizational levels will obtain a high rank-order similarity (i.e., weak metric isomorphic condition), but they will not satisfy the full (or partial) strong metric isomorphic condition. The individual- and organizational-level stressors are similarly defined but we might expect that differing combinations of top-down and bottom-up processes will be reflected in the strength of the relationship between the latent constructs and their target indicators.

### Alternative Multilevel Factor Models of the Management Standards Indicator Tool

Although the factor structure in Hypothesis 1 reflects the factor structure implied in the design of the tool and it was found in Edwards et al.’s (2008) evaluation and confirmed in the Italian context (Rondinone et al., 2012), there may be other plausible and meaningful multilevel-factor models fitting the observed data. For example, data may conform to a multilevel factor model akin to van Veldhoven et al.’s (2005) parsimonious model which represents the Karasek’s triad at both levels, or to a different factor structure across the two levels. Analogous to this, Tay et al. (2014) provide the example that two facets of job satisfaction, task and co-worker satisfaction, may be discrete at the individual level but converge into one factor at the country level.

There are two ways of conjecturing how a three-factor model might be intelligible. The most obvious starting point for such an alternative model is one with the following elements: (1) peer and managerial support are not discrete, (2) the support for change items are concerned primarily with how supportive management is in change situations, as opposed to, for example, how much employees participate in the design of changes; (3) the role items are about clarity and reducing uncertainty, which in themselves are additional forms of support, and (4) the relationship items which are dominated by conflict may represent a form of emotional demands, and may therefore be part of a demands factor. We thus test whether a three-factor multilevel model fits better than that implied by Hypothesis 1:

Hypothesis 2a: *A multilevel confirmatory factor model positing three factors – demands (demands and relationship items), control (control items), and support (peer support, management support, support for change, and role clarity items) – at both levels of analysis* (Model 2a) *will not fit worse the data than Model 1.*

An alternative to this might be: (a) that role clarity is a form of demand, as its achievement amounts to a (bureaucratic) form of instruction to employees on what their job entails and how it should be done, meaning that role clarity items load on demands, and (b) that the relationship conflict might reflect unsupportive behavior, and hence load on a support factor. In this case, a three-factor model may still fit, but the nature of the three factors differs from Hypothesis 2a. Thus, we may hypothesize that:

Hypothesis 2b: *A multilevel confirmatory factor model positing three factors – demands (demands and role clarity items), control (control items), and support (peer support, management support, support for change, and relationship items) – at both levels of analysis* (Model 2b) *will not fit worse the data than Model 1.*

An alternative to the three-factor model – and a distinct possibility given the success of past attempts to fit a single-level seven-factor model to the individual-level data – is that the three-factor structure fits the organizational level whereas a seven-factor structure fits the individual level and vice-versa. We thus further test these two alternative models, hypothesizing that:

Hypothesis 3a*: A multilevel confirmatory factor model positing three factors at the* organization*al level – demands (demands and relationship items), control (control items), and support (peer support, management support, support for change, and role clarity items), and seven factors specified at the individual level* (Model 3a) *will not fit worse the data than Model 1.*

Hypothesis 3b*: A multilevel confirmatory factor model positing three factors at the* organization*al level – demands (demands, and role clarity items), control (control items), and support (peer support, management support, support for change, and relationship items), and seven factors at the individual level* (Model 3b) *will not fit worse the data than Model 1.*

An alternative model was formulated on the basis of the previous single-level analysis of the Management Standards Indicator Tool which found that peer support, management support, and support for change were strongly correlated (Edwards et al., 2008; Rondinone et al., 2012; Toderi et al., 2013). This suggests that the common variance among the support items might be more efficiently explained by a single factor. We thus tested the following two additional models:

Hypothesis 4a*: A multilevel confirmatory factor model positing five factors both at the individual and the* organization*al level* (Model 4a) *– demands, control, relationships, role clarity, and support (peer support, management support, support for change items) will not fit worse the data than Model 1.*

Hypothesis 4b*: A multilevel confirmatory factor model positing five factors at the* organization*al level – demands, control, relationships, role clarity, and support (peer support, management support, support for change items) and seven factors specified at the individual level* (Model 4b) *will not fit worse the data than Model 1.*

A final model we consider is one that is consistent with the attempts to develop dichotomous classifications of organizations, such as the Theory X and Theory Y contrast, which implies a monodimensional factor structure at the organizational level. In particular, we hypothesize that a single organizational latent dimension in which the support items load more strongly than others on the factor reflects a unidimensional concept akin to Theory Y.

Hypothesis 5*: A multilevel confirmatory factor model positing one factor at the* organization*al level and seven factors at the individual level* (Model 5) *will not fit worse the data than Model 1.*

The study we now report is designed to test our hypotheses by using a large sample of multilevel data collected by INAIL. It enables us to test the degree of isomorphism between the organizational- and job-level measures and the extent to which the implied seven-factor model implied by the HSE approach is the best one.

## Materials and Methods

### Procedure and Data Collection

The data used are extracted from INAIL’s web platform that allows organizations to complete a two-stage assessment (i.e., preliminary and in-depth stages) of psychosocial risk factors for work-related stress (Istituto Nazionale Infortuni Sul Lavoro [INAIL], 2013). Our tests of isomorphism use the data gained from employees as part of the in-depth phase, which is collected through the Italian translation of the HSE Management Standards Indicator Tool (Toderi et al., 2013). For a criterion validity test, we used data acquired through the preliminary assessment, using INAIL’s checklist (Istituto Nazionale Infortuni Sul Lavoro [INAIL], 2013), which is completed by a steering group composed of the employer, its representative or occupational health and safety professionals.

Istituto Nazionale Infortuni Sul Lavoro [INAIL] (2013) recommends organizations to use their tools for both the preliminary and in-depth stages, and uploading the data into the web platform. The assessment of work-related stress factors is mandatory for the Italian legal requirements, the adoption of the INAIL’s methodology is not, however, compulsory for organizations. Consequently, only a subsample of the organizations (36%) have completed both stages, so while for the test of isomorphism we use the overall sample, this subsample will be used for the validation exercise.

### Ethics Statement

This study uses data collected within the assessment procedure for work-related stress factors, which are stored within INAIL’s system. This is only accessible to participating organization and, even then, this is limited to that on their organization. INAIL made the data available to the researchers involved in this study without the names of the organizations. Prior to the data collection process, organizations were informed through their management about INAIL’s aims and the potential uses of the data and the security provisions. They were also asked to complete an informed consent form to allow INAIL to permit the use of aggregated anonymized data for research purposes, such as the ones in this study. As this research is based on secondary analysis of anonymized data provided by INAIL, approval from the ethical committee of the academic institutions involved in this study is not needed.

### Sample

The sample of organizations comprises those whose employees completed the INAIL’s in-depth assessment between May 2011 and March 2016. We excluded employees with missing values on more than one-third of the 35 items. The sample for this study consisted of 66,188 employees nested within 775 organizations (the average number of employees participating in INAIL’s in-depth assessment per organization = 85.40, *SD* = 221.66). The proportion of females in the sample was 52.9%. Respondents under 30 years old represented 9.3% of the sample, 60% were between 30 and 50 years old, and less than one-third were over 50 years old. Italian respondents were 94.9%, and the rest of the sample spoke fluent Italian. A large majority of employees had a permanent job (88.9%), while 7.2% had a fixed-term contract, 2.2% were agency workers, and the rest of the sample (1.6%) had temporary or other forms of job contract.

The organizations in the sample were from all industrial sectors: 19.5% in healthcare and social welfare; 18.3% in manufacturing; 13% in professional, scientific, and technical activities; 11.6% in public administration and services; 5.2% in construction; 5.2% in wholesale, retail, or accommodation; 3.2% in transportation and storage; 3.1% in information and communication, financial, and insurance activities and real estate; and 0.9% in agriculture and forestry. The rest of the organizations (19.9%) were involved in other service activities.

The subsample of organizations that completed both phases of data collection used in our validation exercise consists of 19,939 employees nested within 279 organizations. This represents 30% of the individuals and 36% of the organizations constituting our original sample. Tests comparing this subsample with the main sample in terms of their industrial sector and organizational size showed it to be representative of the main sample. There are some differences in the composition of individuals: males (χ^2^_[__1__]_ = 618.02, *p* < 0.001), employees aged 51 or over (χ^2^_[__2__]_ = 536.26, *p* < 0.001) and temporary workers (χ^2^_[__4__]_ = 38.45, *p* < 0.001) are overrepresented, while fixed-term employers underrepresented. There was no difference in the distribution of the nationalities or occupations of the employees.

### Measures

#### Psychosocial Risk Factors for Work-Related Stress

These are measured by the 35 items that comprise the Management Standards Indicator Tool (see Appendix 1), which cover seven dimensions: *Demands* (i.e., workload, work patterns, and work environment, eight items, sample item “I have unachievable deadlines”), *Control* (i.e., control that the employee can exercise on his/her own work activities, six items, sample item “I have a choice in deciding how I do my work”), *Managerial support* (i.e., encouragement and support provided by the employer and management to employees, five items, sample item “I am given supportive feedback on the work I do”), *Peer support* (i.e., encouragement and support provided by colleagues, four items, sample item “I get the help and support I need from colleagues”), *Relationships* (i.e., perceptions of interpersonal conflict at work, four items, sample item “Relationships at work are strained”), *Role clarity* (i.e., understanding of the employee’s own role to avoid role conflict, five items, sample item “I am clear what my duties and responsibilities are”), *Support for change* (i.e., organizational change processes and how changes are communicated, three items, sample item “I have sufficient opportunities to question managers about change at work”). Following Edwards et al. (2008), Health and Safety Executive [HSE] (2019), employees were asked to answer statements on five-point Likert-type response scales, which varied according to the type of question: frequency ones using from 1 = *never* to 5 = *always* (for items 1–23) and agreement ones from 1 = *strongly disagree*, 5 = *strongly agree* (for items 24–35). Employees were asked to refer to the time frame of the 6 months prior to the questionnaire administration when providing their evaluations. All items are scored so that lower scores reflect higher levels of psychosocial risk (see Appendix 1). Thus, demands and relationships items were reversely coded so that lower scores reflect higher risk. This approach to coding was originally devised by the British creators of the HSE approach (see Cousins et al., 2004; Edwards et al., 2008) as it “helps comparison across the […] factors” (Edwards et al., 2008, p. 102).

#### Organizational Indicators of Work-Related Stress

Three items from the INAIL checklist, acquired in the preliminary INAIL’s assessment (Barbaranelli et al., 2018), were used in the criterion validation: injuries, sickness absence, and grievances over stress and health issues. Organizational steering groups were asked to use objective organizational recorded data and indicate whether *work-related injuries* and *sickness absences* had “decreased,” remained “static” or “increased” over the last 3 years (respectively, coded as 0, 1, and 2) and whether there are any *grievances over stress and health* at the time of the assessment (no coded as 0 and yes as 1).

### Analytic Strategy

We first assessed whether there is a need for multilevel modeling by evaluating the intraclass correlation coefficient (ICC) and the design effect, or *deff* (Muthén and Satorra, 1995; Hox, 2010). The ICC refers to the proportion of item variability that exists at the organizational level, and the *deff* is an index that quantifies how the sampling error under the nested structure of the data departs from the sampling error under simple random sampling (Heck and Thomas, 2015). ICC values > 0.10 (Bliese, 2000) and a *deff* greater than 2 can be considered as indicative of non-trivial clustering effects that should be taken into account in analyzing the data (Muthén and Satorra, 1995). We also examined the within-organization agreement by means of the rwg_(_*_*j*_*_)_ at the item level (Biemann et al., 2012) assuming a rectangular distribution of agreement. Values above 0.71 indicate strong within-organization agreement, while those between 0.51 and 0.70 indicate moderate agreement.

Once the need for multilevel modeling was established, we tested the models implied by Hypotheses 1–5 through multilevel confirmatory factor analysis. These were tested in M*plus* 8 (Muthén and Muthén, 1998–2017), using maximum likelihood estimation with standard errors based on first-order derivatives (abbreviated as MLF in M*plus*) and the full information maximum likelihood approach to handling missing data (Arbuckle, 1996). To assess the overall goodness of fit of the hypothesized models, we used several indices: (1) Chi-squared test; (2) root-mean-square error of approximation (RMSEA) (Steiger, 1990); (3) comparative fit index (CFI) (Bentler, 1990); (4) Tucker–Lewis index (TLI) (Tucker and Lewis, 1973); and (5) standardized root-mean-squared residual (SRMR) (Hu and Bentler, 1999). Since not all the alternative models we tested were nested in Model 1, they were compared by means of the expected cross-validation index (ECVI; Browne and Cudeck, 1993) and Akaike information criterion (Akaike, 1973). The model with the lowest EVCI and AIC is considered the best fitting.

If the best fitting model has the same factor structure at both levels of analysis (i.e., strong configural isomorphic condition), tests for strong metric isomorphism will be conducted by constraining the same factor loadings to equality across levels. If the Δχ^2^_(__Δ__df)_ between the strong metric model and the unconstrained model is not significant for *p* < 0.01 (Scott-Lennox and Lennox, 1995), strong metric isomorphism can be assumed. If strong metric isomorphism is not reached, partial strong metric isomorphism will be tested by releasing one-by-one the equality constraints on factor loadings across levels on the basis of modification indices (Tay et al., 2014), and the same statistical test [Δχ^2^_(__Δ__df__)_] will be repeated to determine whether there is partial metric strong isomorphism. Similarly, if partial strong metric isomorphism is not supported by the data, weak metric isomorphism will be evaluated via the similarity in the rank ordering of factor loadings across levels. According to Tay et al. (2014), the weak metric isomorphic condition is satisfied when the rank-order correlation among individual- and organizational-level factor loadings is greater than 0.50. Moreover, Tucker *phi* congruence coefficients will be computed for each dimension between individual- and organizational-level loadings. Following Lorenzo-Seva and Ten Berge (2006), values higher than 0.85 suggest good similarity, while values approaching 0.95 indicate a high similarity among factor loadings of a given latent dimension.

Multilevel reliability of the constructs posited by the hypothesized model was examined using three indices provided for each level (see Geldhof et al., 2014): (1) two-level internal consistency (α); (2) two-level composite reliability (ω); and (3) two-level maximal reliability (H). While the α coefficient represents the average of inter-item correlations, ω and H are more trustworthy reliability estimates of the true total score. Although cut-off values of these indices for multilevel factor analysis have not yet been proposed, relying on classical test theory we consider acceptable values to be those greater than 0.70 (Raykov and Marcoulides, 2011).

Finally, we derived organizational-level factor scores from the best fitting model and used them as dependent variables for assessing criterion validity. First, we assessed a one-way MANOVA evaluating the extent of multivariate and univariate differences in the factor scores among industries by means of Duncan *post hoc* tests, which controls for Type II error. Second, we investigated the relationship between our organizational-level factors and the three organizational stress indicators using polyserial correlations for the injuries and absence indicators and biserial correlations for grievances. Third, we considered the correlation between the stressor factors and organizational size.

## Results

Examination of the descriptive statistics of the Management Standards Indicator Tool items revealed that some of them are not normally distributed, specifically items 1, 4, 5, 11, 13, 18, and 21 (Appendix 1). Since MLF estimators are not robust to non-normality (see Muthén and Muthén, 1998–2017), we applied log10 transformations to these items (Tabachnick and Fidell, 2013). After this adjustment, their skewness ranged from −0.50 to −0.91, while kurtosis ranged from −1.24 to −0.36. The ICCs ranged from 0.14 to 0.28 (*M* = 0.19, *SD* = 0.04), and *deff* values were between 11.648 and 23.886. Rwg_(_*_*j*_*_)_ indices ranged between 0.78 and 0.96, suggesting a strong degree of within-organization agreement on the items. These results suggest that the nested structure of the data cannot be ignored and multilevel factor analysis is required.

The results for the models implied in Hypotheses 1–5 are presented in Table 1. All alternative multilevel confirmatory factor models fit the data worse than Model 1, which posits the same seven-factor structure and the same pattern of free and fixed factor loadings at both the individual and the organizational level. Thus, strong configural isomorphism criteria were satisfied. Then, we tested whether the seven-factor multilevel model met the strong metric isomorphism criteria and found that it did not [Δχ^2^_(Δdf = 35)_ = 940.16, *p* < 0.001]. Moreover, when releasing one-by-one the equality constraints on factor loadings across levels, as suggested by modification indices (see Millsap, 2011), partial strong metric isomorphism was not found for any factor.

**TABLE 1:** Fit indices of the alternative multilevel confirmatory factor models of the Management Standards Indicator Tool.

**Referent hypothesis**	**MLF-based χ^2^**	**df**	***p***	**RMSEA**	**CFI**	**TLI**	**SRMR within**	**SRMR between**	**ECVI**	**AIC**
H1	96,193.18	1078	<0.001	0.037	0.911	0.902	0.048	0.097	124.84	4,309,696.66
H2a	225,090.13	1114	<0.001	0.055	0.836	0.817	0.070	0.107	291.28	4,438,521.61
H2b	248,461.98	1114	<0.001	0.058	0.815	0.794	0.076	0.228	321.48	4,461,893.46
H3a	220,854.56	1096	<0.001	0.055	0.794	0.772	0.070	0.104	285.85	4,434,321.60
H3b	243,517.49	1096	<0.001	0.058	0.748	0.745	0.076	0.109	315.14	4,456,984.45
H4a	142,010.03	1100	<0.001	0.044	0.874	0.860	0.054	0.258	127.33	4,355,469.50
H4b	96,783.179	1089	<0.001	0.036	0.910	0.900	0.048	0.098	125.57	4,310,812.64
H5	100,524.123	1099	<0.001	0.037	0.906	0.899	0.048	0.152	130.38	4,313,985.60

*MLF-based χ^2^ = Chi-square associated with the maximum likelihood estimation function with standard errors based on the first-order derivatives; *df* = degrees of freedom; RMSEA = root-mean-square error of approximation; CFI = comparative fit index; TLI = Tucker–Lewis or non-normed fit index; SRMR = standardized root-mean-square residual (*within* is referred to the individual level, *between* is referred to the organizational level).*

Consequently, we tested for weak metric isomorphism, and found that the rank-order correlation coefficient between individual- and organizational-level factor loadings was 0.85, while Tucker *phi* congruence coefficients ranged from 0.99 (for relationships) to 1 (for support for change). Overall, these results suggest that the factor loadings are highly congruent across levels, and weak metric isomorphism between the individual and organizational measures has been found for all seven factors.

The vast majority of factor loadings in the seven-factor model are above 0.90 at the organizational level and above 0.50 at the individual level (Table 2). The factor loadings at the organizational level are consistently higher than those at the individual level, a common finding in multilevel factor analysis (Kim et al., 2016). Factor correlations at the individual level are consistent with Edwards et al.’s (2008) original results, while at the organizational level, correlations among managerial support, peer support, and support for change are particularly high (Table 3). Nonetheless, following Can et al. (2015, p. 420), we view the “constructs … as conceptually different as long as there is not a correlation of 1 between them.” The reliability indices for the individual and organizational dimensions are satisfactory for all factors at both levels, although those for the organizational-level factors are systematically higher than those for the individual-level factors.

**TABLE 2:** Standardized factor loadings from the seven-factor model.

***Item***	**Demands**	***Item***	**Control**	***Item***	**Managerial support**	***Item***	**Peer support**
*3*	0.560 (0.906)	*2*	0.461 (0.725)	*8*	0.580 (0.903)	*7*	0.673 (0.938)
*6*	0.693 (0.944)	*10*	0.676 (0.942)	*23*	0.761 (0.964)	*24*	0.815 (0.977)
*9*	0.452 (0.554)	*15*	0.753 (0.957)	*29*	0.779 (0.960)	*27*	0.708 (0.935)
*12*	0.615 (0.912)	*19*	0.692 (0.906)	*33*	0.621 (0.963)	*31*	0.745 (0.969)
*16*	0.418 (0.730)	*25*	0.718 (0.925)	*35*	0.765 (0.926)		
*18*	0.488 (0.791)	*30*	0.387 (0.641)				
*20*	0.543 (0.626)						
*22*	0.744 (0.960)						

***Item***	**Relationships**	***Item***	**Role clarity**	***Item***	**Support for change**		

*5*	0.728 (0.979)	*1*	0.658 (0.951)	*26*	0.748 (0.961)		
*14*	0.602 (0.874)	*4*	0.473 (0.877)	*28*	0.715 (0.932)		
*21*	0.765 (0.966)	*11*	0.668 (0.940)	*32*	0.648 (0.951)		
*34*	0.438 (0.886)	*13*	0.766 (0.965)				
		*17*	0.712 (0.922)				

*Values not in brackets refer to the individual level and values within brackets refer to the organizational level.*

**TABLE 3:** Multilevel reliability indices and correlations at individual and organizational levels.

	**Reliability**			**Correlations**						
		
	**α**	**ω**	**H**	**1.**	**2.**	**3.**	**4.**	**5.**	**6.**	**7.**
1. Demands	0.78 (0.93)	0.79 (0.94)	0.82 (0.97)	–	0.49	0.69	0.61	0.81	0.60	0.68
2. Control	0.76 (0.92)	0.79 (0.94)	0.82 (0.97)	0.35	–	0.68	0.71	0.54	0.48	0.67
3. Managerial support	0.82 (0.97)	0.83 (0.97)	0.84 (0.98)	0.41	0.54	–	0.91	0.75	0.74	0.95
4. Peer support	0.82 (0.97)	0.82 (0.98)	0.83 (0.98)	0.34	0.49	0.67	–	0.75	0.71	0.82
5. Relationships	0.72 (0.96)	0.73 (0.96)	0.77 (0.98)	0.56	0.44	0.58	0.60	–	0.52	0.65
6. Role clarity	0.79 (0.97)	0.79 (0.97)	0.81 (0.97)	0.32	0.48	0.54	0.42	0.38	–	0.72
7. Support for change	0.74 (0.96)	0.74 (0.96)	0.75 (0.96)	0.42	0.62	0.85	0.62	0.52	0.64	–

*α = Cronbach’s alpha; ω = composite reliability; H = maximal reliability. Since the linear transformation applied to some items (e.g., log10) changed their variances, α is based on inter-item correlations (i.e., standardized α, see Falk and Savalei, 2011). For reliability, values not in brackets refer to the individual level and values within brackets refer to the organizational level. Correlations below the diagonal refer to the individual level, while those above the diagonal refer to the organizational level. All latent correlations are significant for *p* < 0.001.*

Overall, strong configural and weak metric isomorphism between the organizational and individual level were supported by the observed data, but this was not the case for the strong metric isomorphism for any factor. The seven-factor model (Hypothesis 1) was found to fit best when compared with more parsimonious models (Hypotheses 2a–5). Thus, we can conclude that in the present sample, the number of factors is the same across levels with the same pattern of fixed and free factor loadings, and the magnitude of factor loadings has the same rank order across levels, but the factor loadings are not identical across levels.

### Criterion Validity

Our first test for criterion validity revealed that the Management Standards Indicator Tool organizational-level factor scores varied significantly across industrial sectors. There was a large multivariate significant effect [*F*_(__63_,_1487__)_ = 3.787, *p* < 0.001; Wilks’ Λ = 0.433, partial η^2^ = 0.113]. All principal effects were significant and showed high effect sizes (Table 4). *Post hoc* tests revealed differences across the factors. High demands and low role clarity were more relevant risk factors in the information, scientific, and technical sectors than in the agriculture and construction sectors. Low control and low relationships were more relevant risk factors in the transportation sector than in the wholesale, construction, and agriculture sectors. Managerial support was a less important risk factor in the construction sector than in any other, while poor peer support and support for change were more relevant risk factors in the agriculture and construction sectors than in any other except wholesale. Notably, the three most highly correlated factors – managerial support, peer support, and support for change – did not share the same pattern across industrial sectors. This adds support to the conclusion that the seven factors are discrete.

**TABLE 4:** Management standards dimensions by industry.

	***F*_(__9_,_269__)_^∗^**	**partial η^2^**	**Agriculture, forestry, and fishing (*n* = 2)**	**Manufacturing (*n* = 72)**	**Construction (*n* = 25)**	**Wholesale, retail trade, accommodation, and food service activities (*n* = 15)**	**Transportation and storage (*n* = 10)**	**Information and communication, financial and insurance activities, real estate (*n* = 14)**	**Professional, scientific, and technical activities (*n* = 44)**	**Education, public administration and defense, compulsory social security (*n* = 35)**	**Human health and social work activities (*n* = 49)**	**Other service activities (*n* = 13)**
Demands	6.625	0.181	–		–		–	+	+			
Control	4.774	0.138	–		–	–	+				+	
Managerial support	6.995	0.190	+	+	–	+	+	+	+	+	+	+
Peer support	5.608	0.158	–	+	–		+	+	+	+	+	+
Relationships	7.450	0.200	+	+	–	+	+	+	+	+	+	+
Role Clarity	4.433	0.129	–		–		–	+	+			
Support for change	6.657	0.182	–	+	–		+	+	+	+	+	+

*^∗^All the tests were significant for *p* < 0.001. Symbol “−” indicates that the mean of the economic sector is significantly lower than those indicated by “+,” as indicated by Duncan *post hoc* tests, where higher scores reflect higher risk for work-related stress.*

Our second validity test revealed that most of the correlations among organizational-level factor scores and organizational stress indicators were significantly different from zero (Table 5). All dimensions were significantly associated with the injuries, sickness absences, and grievances indicators, with three (out of 21) exceptions, as role clarity was unrelated to either absence or grievances, and control was unrelated to grievances. The magnitude of correlations varied from low to moderate. Work-related injuries, sickness absences, and grievances were then more likely to have increased in the past 3 years in organizations scoring highly on the dimensions of our measure of organizational-level stressors, notwithstanding the three exceptional cases. Finally, all organizational factors, apart from role clarity, were associated with organizational size. Rank-order correlation coefficients (all statistically significant at *p* < 0.001) were 0.23 for demands, 0.23 for control, 0.23 for management support, 0.13 for peer support, 0.21 for relationships, and 0.27 for support for change. These results suggest that the level of stressors is greater in bigger organizations, excepting for role clarity. They are consistent with past research on stress factors (e.g., Dekker and Barling, 1995) and rewards (e.g., Ingham, 1970) and findings that stress is positively related to organizational size (Health and Safety Executive [HSE], 2016) and job satisfaction is negatively related to organizational size (Clark et al., 1996; Bender et al., 2005).

**TABLE 5:** Correlations of management standards dimensions with organizational indicators of work-related stress.

	**Work-related injuries polyserial correlations**	**Sickness absences polyserial correlations**	**Grievances over stress and health biserial correlations**
1. Demands	0.185^∗∗^	0.167^∗^	0.345^∗∗∗^
2. Control	0.148^∗^	0.178^∗∗^	0.145^ns^
3. Managerial support	0.203^∗∗^	0.169^∗^	0.290^∗∗^
4. Peer support	0.193^∗∗^	0.169^∗^	0.203^∗^
5. Relationships	0.229^∗∗∗^	0.209^∗∗^	0.345^∗∗∗^
6. Role clarity	0.140^∗^	0.120^ns^	0.120^ns^
7. Support for change	0.189^∗∗^	0.167^∗^	0.294^∗∗^

*ns = not significant, ^∗^*p* < 0.05, ^∗∗^*p* < 0.01, ^∗∗∗^*p* < 0.001.*

## Discussion

The aim of this research was to assess whether a measure of organizational-level stressors could be derived from data on job characteristics collected from individual employees through the Management Standards Indicator Tool. Using multilevel confirmatory factor analysis, the seven-factor oblique factor structure mirroring the architects’ design, and originally tested at the individual level by Edwards et al. (2008), was tested simultaneously at the individual and organizational levels and we found the model fitted the observed data and moreover was better than some other possible models. Thus, the model of Hypothesis 1 is more supported by the data than those of Hypotheses 2a–5. This outcome provides evidence for the strong configural isomorphism of the Management Standards Indicator Tool, that is, the same number of factors with the same pattern of free- and fixed-factor loading fitted the observed data at both levels. Moreover, while strong metric isomorphism (full or partial) was not found, the weak metric isomorphic condition was satisfied.

Our results confirmed the feasibility of achieving the intentions of architects of the Management Standards Indicator Tool that the individual-level data can be used to develop measures of organizational-level stressors, in addition to its provision of a measure of job-level stressors. The weak metric isomorphism means that the organizational-level stressors are not simply the mirror of individual-level job stressors and that individuals’ experiences of these are not interchangeable for determining measures at the organizational level (Stapleton et al., 2016). In terms of Rousseau’s (1985) distinction between levels of measurement and levels of analysis, the level of measurement is the individual level and the object of measurement is at the job level, identified primarily on the basis of the extended Karasek theory of stress. The ambition behind the Management Standards Indicator Tool is based on a multilevel theory of work-related stress that it may be a result of two types of work-related stressors: organizational-level and job-level ones. Our results offer support for the theoretical assumptions underlying it and in particular confirm that the organizational-level stressors are identifiable phenomena and thus including these in analysis of individual, group, or organizational stress levels is meaningful. For policy, targeting organizational-wide interventions at these may be fruitful either alone or in conjunction with job design or helping individuals reduce their internal sources of stress and ability to cope with external stressors.

The extent to which weak metric isomorphism reflects the combination and nature of top-down and bottom-up processes cannot be gauged from such results. It is a matter of theory, as is ascertaining the precise ontological status of the higher-level constructs, as Tay et al. (2014) stress. In treating our organizational factors as organizational-level phenomena, we are according them a “collective” ontological status. Organizational psychosocial risk factors for work-related stress cannot then be conceptualized as the average employees’ experience within an organization, since the within-organization variability of this among employees should be taken into account when basing measures of organizational-level stressors on measures of this. Only in the extreme case that the individual- and organizational-level loadings were identical and the residual variances of the manifest indicators of the organizational level were zero (Jak et al., 2013), could the average employees’ experience meaningfully represent the organizational level. This extreme situation would be consistent with a holistic view of an organization founded on methodological individualism in which individual actions are the basis of all social phenomena, even though individuals’ actions depend on their social relations with each other (Tollefsen, 2014).

The concept of a collective, in contrast, implies some level of shared intentionality (Tollefsen, 2014). As such, our organizational-level measure can be treated as reflecting the orientation toward specific psychosocial risks for work-related stress in the organization, and as a good guide to the state of the risks within it, as the HSE initially intended. The extent to which the intentionality underlying the level of organizational stressors reflects goals concerning safety, health, and well-being, or managers’ representations of the ideal working environment, will vary. Such high stress-consciousness or prioritization of employees’ well-being is only likely to exist in organizations at lower ends of any risk for work-related stress. At the higher ends, managements may not be particularly conscious of the dangers of stress or even of the well-being of their workforce and the intentionalities creating risky conditions for work-related stress may be dominated by the drive for productivity and other economic exigencies.

### Implications for Research

The items in the Management Standard Indicator Tool assess employee’s experience of several job characteristics within the work environment (i.e., the direct consensus approach; see Chan, 1998). It is not uncommon to use individual scores to create organizational aggregates by summing or averaging them within organizations (Chen et al., 2005), but our results indicate the need to use organizational-level factor scores from the strong configural model because the analysis takes into account the within-organization variability around the point of convergence, that is the shared element of employees’ responses (Bliese, 2000; Heck and Thomas, 2015). Moreover, the multilevel factor analytic approach allows one to interpret individual-level factor scores as partialed out from contextual influences and as reflecting the job-level dimension of work-related stressors, after controlling for the differences attributable to the organizational level.

The added value of the present study to the work-related stress field is that it facilitates and, to put it more strongly, demands the inclusion of both organizational-and job-level risk factors into individual-level models of work-related stress and how these interact with each other, as well as perhaps individual-level stressors such as neuroticism, pessimism, or inadequate coping styles. Both measures of stressors may also be used to test and develop theories that include both effects on such organizational phenomena as absenteeism, abuse, discrimination, and employee health. Both are also invaluable for testing models of collective stress and other such outcomes.

The measure of organizational-level stressors may also be used to compare organizations, establish industrial- and national-level multi-dimensional benchmarks, and assess the location and determinants of organizations with particular profiles of risk for work-related stress. More specifically, even though some of the organizational-level dimensions are highly correlated, the organizational-level scores may be used for profiling or identifying categories of organizations. Anchoring an organization-centered analysis in our variable-centered approach helps to achieve more precise profiles (Morin et al., 2017). For example, a group of organizations may be identified that has poor peer support, low managerial support, and medium support for change. Such configurations can then be used to establish and compare the distribution of stressors across the economy at the individual and organizational levels. Such an exercise would also be useful for identifying cases for further investigation. These might include cross-level analysis of misfits (Chan, 1998) such as organizations where a sizeable proportion of individuals face high levels of risk factors on at least some dimensions as identified by the individual-level factor scores, yet the organizational-level measures reveal a relatively low risk for work-related stress; or alternatively cases where individuals do not have high levels of risk for work-related stress but work in high-risk organizations. Further research could then link the analysis of stressors to actual levels of stress and health (as Bevan et al., 2010 have done using HSE data) and assess whether certain combinations of risk factors are disproportionately associated with high or low levels of stress within the organization. For example, if a lower level of support from one’s manager has a greater impact on ill-being when either the level of organizational stressors is high or, the overall level of such support in an organization is high (i.e., there is an outsider effect).

It is also tempting to suggest that further validation of the measure in other countries would be fruitful. However, given the success of our validation and high levels of effort and time required to collect such data on the scale INAIL has done, it may be that this would be of only marginal benefit. On the other hand, if other national bodies follow INAIL’s example, such datasets may become more commonplace. Further testing of the criterion validity of our measures with stronger measures of well-being, accidents, and absenteeism would be especially valuable, and would enable exploration of the extent of homology across the individual and organizational levels.

### Implications for Practice

The main policy implication of this research is that the Management Standards Indicator Tool can be used as originally intended by its architects. Our study completes the fulfillment of the desire of the creators of the Management Standards approach that it should be evidence-based. The direct consensus method of composition has been shown to be effective. The strategy of using individual respondents to assess an organization’s risk of stressfulness on the basis of their experience of psychosocial risk factors associated with their jobs – rather than asking directly about the organization or using key informants – is valid.

Moreover, in establishing the meaningfulness and the ontological status of the concept of organizational-level stressors or psychosocial risk factors for work-related stress, our study adds to the conviction of the HSE, INAIL, and others, that organization-wide interventions aimed at reducing work-related stress should be encouraged and may reap benefits alongside those conducted at the job level. Both HSE and INAIL provide in their methodologies a classification of each organizational factor of the Management Standards Indicator Tool based on four levels of risks based on percentiles, which is applied directly to the average scores across employees within each organization. We, however, recommend creating norms and cut-offs to organizational-level factor scores derived from the strong configural multilevel factor model, which can be calculated from organizational raw aggregates (Gorsuch, 1983), in order to control for individual-level differences, random and unique measurement error (Heck and Thomas, 2015). Moreover, norms should be also differentiated by economic sectors, which would allow a more precise classification of organizational-level risks.

The HSE developed the Management Standards Indicator Tool as part of a wider approach to helping organizations to fulfill their legal obligations to provide a safe environment for employees and encourage them to acknowledge that organizational factors can be a powerful source of work-related stress. It offered a step-by-step approach that involved strong senior management involvement, project planning, ensuring adequate resources for developing projects and maintaining processes, and strategies for data collection and communication. A variety of data and collection methods may be used, including focus groups and routinely collected data (e.g., on absence and labor turnover), but Health and Safety Executive [HSE] (2009) prioritizes surveys, especially for large or multi-site organizations, and the use of a multidimensional indicator tool for these. The HSE provides access to the instrument via its website but INAIL, uniquely, enables organizations to use the web site to upload the data they generate from administering the indicator tool to INAIL’s central repository. This, augmented by data from specific projects and collaborative research projects, has enabled INAIL to create the national dataset from which our data were acquired (Istituto Nazionale Infortuni Sul Lavoro [INAIL], 2013; Persechino et al., 2013). This dataset also includes the stress indicators collected using organizational objective data, acquired in the preliminary assessment stage of INAIL’s recommended procedure and used in our validation exercise. The dataset is structured on two layers, by industry and organization, and this adds to its uniqueness.

Such a comprehensive adaptation of the HSE standards approach could be followed by other countries, and our validation of the Management Standards instrument’s ability to capture its desired organizational-level perspective adds credence to the value of such an initiative. Validated measures of psychosocial risk factors for work-related stress can aid public bodies like the United States’ National Institute of Occupational Safety and Health who wish to encourage health and safety interventions and research on the “factors influencing the motivation and capacity of firms to implement organizational interventions” (National Institute of Occupational Safety and Health [NIOSH], 2002, p. vii). The implication of our results is that such public agencies should continue to go beyond blanket exhortation of the dangers of stress. They should encourage organizations to use the dimensions of the organizational risk for work-related stress in the HSE framework as a language to discuss and identify potential stress points and use the indicator tool to measure their risk of stress and the metrics to design interventions that are targeted where the greatest risks lie.

The implications for organizations mirror those for national bodies, as the research suggests they should investigate their work organizations and existing policies and procedures, the degree to which these generate an at-risk environment for work-related stress, and which risk factors are most relevant in their organization. Having such a precise assessment of the organization’s stressors allows management to then establish the most effective corrective and preventive interventions with the aim of improving employees’ health and safety and enhancing productivity (Nielsen and Randall, 2013). The tool is relevant whether an organization has a high or low level of stressfulness – in either case, management can discover where they are failing or succeeding, and hence where they need to improve or to avoid being complacent.

While the underlying Management Standards approach is an example of an evidence-based approach to management, which encourages an empirical-rational approach to change (Chin and Benne, 1969), it does not rule out the involvement of employees and their representatives in its implementation or the design of interventions. As in the survey-feedback method of attitude surveying, participative methods can be used in the discussion of the scores on the organizational-level (and individual-level) measures of stressors and then in the construction and implementation of interventions. Several studies have indicated that the active involvement of employees plays a key role in the success of organizational interventions for health (Nielsen and Randall, 2013), not least as employee participation can contribute to the correct estimation of risk as employees are an essential source of information about their working conditions, as our study illustrates.

### Limitations of the Study and Future Directions

The strength of the study is that it is based on a large dataset both in terms of surveyed employees and organizations, and we used the latest statistical technology to test our hypotheses. Nonetheless, the limitations are that the data are cross-sectional, and that we did not considered a statistically representative sample of the Italian population of organizations. Future studies rooted within the multilevel perspective should investigate the stability of (and change in) the Management Standards Indicators. They might also address intermediate levels of analysis (e.g., work groups and departments). Although the HSE’s Management Standards approach for the assessment of psychosocial risk for work-related stress is explicitly focused on organizations (Edwards et al., 2008), stressors at intermediate levels may play a role in employees’ stress levels, as well as team-level outcomes and moods (Costa et al., 2014; Martin et al., 2016). In addition, while the ontology and social processes lying behind the emergence of organizational-level stressors are a matter of theory and even philosophy (Tay et al., 2014), empirical work is needed to address what (and how) individual and organizational processes lead these organizational factors to emerge (Kozlowski and Chao, 2012).

Unfortunately, no other predictors and outcomes of Management Standards Indicator Tool dimensions than those we used were available in our dataset at the individual or organizational level. Moreover, the indicators of organizational stress used for criterion validity exercise reflect trends over the past 3 years and they have been collected at the same time of our measures of stressors. Thus, we evaluated them in terms of correlations with the organizational dimensions of the Management Standard Indicator Tool without inferring any causality among them. Future studies should include stress outcomes and other theoretically plausible determinants and moderators of work-related stress, both at the individual and organizational levels, and validation exercises that have more sophisticated measures of organizational stress than we were able to use.

The Management Standard Indicator Tool covers a limited number of risk factors based on the intrinsic aspects of jobs and their immediate (supportive) context. Further studies should investigate the organizational-level stressors considered in other instruments (such as the extrinsic factors included in Warr’s vitamin model and top-level leadership behavior). Comparisons of different methods of data collection and specifically using items involving the referent shift approach (Wallace et al., 2016) would allow a further diagnosis of the validity of Management Standards Indicator Tool dimensions.

## Conclusion

We have shown that Great Britain’s HSE’s strategy of surveying employees about their jobs in order to develop an organizational-level measure of the potential stressfulness of the psychosocial environment within organizations can produce valid results. Previous validation exercises have concentrated on testing a seven-factor model at the individual level, but our use of multilevel factorial analysis has enabled us to demonstrate, using a large dataset of individual assessments nested within organizations, that the organizational-level measure that represents the seven dimensions in the HSE model is metrically isomorphic with the individual-level measure. In Tay et al.’s (2014) terms, it has weak metric isomorphism – i.e., the factor loadings on the items on all dimensions have similar rankings but not identical magnitudes. This means that the organizational level is not a straightforward mirror of the aggregation of the job level, and suggests that the level of stressors in an organization is not simply the result of bottom-up processes, but may also be generated by top-down measures such as organizational policies. The calls for organization-wide interventions and policy changes that do not directly involve the redesign of jobs or initiatives targeted at individuals are then not misplaced and may yet have more effect, not least as their coverage within the organization may be greater than such initiatives, though part of them may entail encouraging local-level changes or job crafting. Our study has then shown that organizational-level stressors are identifiable phenomena to which researchers and practitioners should pay more attention. That it has shown critical stressors can be validly measured by the Management Standards Indicator Tool suggests that this might be a first port of call for those heeding this suggestion; it provides a tool that has great potentiality to help researchers, organizations, and public bodies to identify stress risks and target interventions to these.

## Data Availability Statement

The datasets generated for this study will not be made publicly available. Data collected within the assessment procedure for work-related stress factors are stored within INAIL’s system. Permission to use the data must be made to INAIL.

## Ethics Statement

Ethical review and approval was not required for the study on human participants in accordance with the local legislation and institutional requirements. The participants provided their written informed consent to participate in this study.

## Author Contributions

SW first drafted the manuscript and made revisions to the manuscript in the light of group discussions, and literature searches on the substantive issue and multilevel analysis. VG and CB contributed particularly to the study method and the data analysis. CD and MR contributed to the literature review and to the institutional framework review. RF and MF contributed to the literature review. SI initiated the study and contributed to its design. All authors contributed to the design of the manuscript and the core theoretical issues, and contributed to the writing of the sections “Discussion” and “Conclusion.”

## Conflict of Interest

The authors declare that the research was conducted in the absence of any commercial or financial relationships that could be construed as a potential conflict of interest.

## Footnotes

1 INAIL is a public body supporting organizations in meeting a legal requirement to manage risks for work-related stress that followed the implementation of the European Framework Agreement on work-related stress (European Trade Union Congress, 2004).

2 The Literature search, conducted in November 2018, used Business Source Complete and PsycINFO databases in November 2018.

## Appendix

**TABLE A1:** Descriptive statistics for the Management Standards Indicator Tool Items at the individual level.

	***M***	***SD***	**SKEW**	**KURT**
**Demands**				
3. Different groups at work demand things from me that are hard to combine	3.26	0.96	–0.02	–0.27
6. I have unachievable deadlines	3.94	1.01	–0.72	–0.09
9. I have to work very intensively	2.21	0.92	0.61	0.30
12. I have to neglect some tasks because I have too much to do	3.29	1.00	–0.03	–0.38
16. I am unable to take sufficient breaks	3.57	1.13	–0.47	–0.57
18. I am pressured to work long hours	4.26	1.02	–1.34	1.08
20. I have to work very fast	2.69	1.06	0.27	–0.39
22. I have unrealistic time pressures	3.93	1.03	–0.76	–0.04
**Control**				
2. I can decide when to take a break (R)	3.47	1.20	–0.50	–0.58
10. I have a say in my own work speed	3.35	1.17	–0.47	–0.58
15. I have a choice in deciding how I do my work (R)	3.57	1.11	–0.73	–0.08
19. I have a choice in deciding what I do at work (R)	3.16	1.24	–0.34	–0.90
25. I have some say over the way I work	3.65	0.95	–0.86	0.60
30. My working time can be flexible (R)	3.34	1.23	–0.52	–0.75
**Managerial support**				
8. I am given supportive feedback on the work I do (R)	3.44	1.05	–0.42	–0.36
23. I can rely on my line manager to help me out with a work problem (R)	3.83	1.21	–0.81	–0.33
29. I can talk to my line manager about something that has upset or annoyed me about work R	3.72	1.11	–0.86	0.13
33. I am supported through emotionally demanding work (R)	3.27	1.07	–0.41	–0.41
35. My line manager encourages me at work (R)	3.45	1.16	–0.57	–0.40
**Peer support**				
7. If work gets difficult, my colleagues will help me (R)	3.76	1.07	–0.66	–0.14
24. I get help and support I need from colleagues (R)	3.74	0.91	–0.80	0.74
27. I receive the respect at work I deserve from my colleagues (R)	3.80	0.92	–0.87	0.82
31. My colleagues are willing to listen to my work-related problems (R)	3.61	0.95	–0.72	0.45
**Relationships**				
5. I am subject to personal harassment in the form of unkind words or behavior	4.16	1.05	–1.06	0.25
14. There is friction or anger between colleagues	3.45	1.05	–0.29	–0.40
21. I am subject to bullying at work	4.34	1.00	–1.46	1.35
34. Relationships at work are strained	3.28	1.12	–0.21	–0.70
**Role clarity**				
1. I am clear what is expected of me at work (R)	4.25	0.90	–1.37	1.90
4. I know how to go about getting my job done (R)	4.50	0.66	–1.64	4.56
11. I am clear what my duties and responsibilities are (R)	4.44	0.84	–1.73	3.15
13. I am clear about the goals and objectives for my department (R)	4.05	1.07	–1.11	0.59
17. I understand how my work fits into the overall aim of the organization (R)	3.90	1.09	–0.93	0.22
**Support for change**				
26. I have sufficient opportunities to question managers about change at work (R)	3.33	1.14	–0.51	–0.54
28. Staff are always consulted about change at work (R)	2.98	1.19	–0.12	–0.92
32. When changes are made at work, I am clear how they will work out in practice (R)	3.40	1.02	–0.54	–0.16

**M* = mean; *SD* = standard deviation; SKEW = skewness; KURT = kurtosis. The numbers of the items refer to the order in the questionnaire; items ending with (R) were inversely coded.*
